# Evaluation of Respiratory Conditions in Individuals Undergoing Rapid Maxillary Expansion: A Computational Fluid Dynamics Study

**DOI:** 10.3390/diagnostics15050527

**Published:** 2025-02-21

**Authors:** Arzu Alan, Mehmet Ugurlu, İbrahim Sevki Bayrakdar, Fehmi Gonuldas, Sergio Lucio Pereira de Castro Lopes, Andre Luiz Ferreira Costa, Kaan Orhan

**Affiliations:** 1 Ankara 75th Year Oral and Dental Health Hospital, Ministry of Health, Ankara 06230, Türkiye; arzudogruyol@yahoo.com; 2 Department of Orhtodontic, Faculty of Dentistry, Osmangazi University, Eskisehir 26040, Türkiye; ugurlum@ogu.edu.tr; 3 Department of Dentomaxillofacial Radiology, Faculty of Dentistry, Osmangazi University, Eskisehir 26040, Türkiye; ibrahimsevkibayrakdar@gmail.com; 4 Department of Prosthodontics, Faculty of Dentistry, Ankara University, Ankara 06560, Türkiye; fgonuldas@ankara.edu.tr; 5 Department of Diagnosis and Surgery, São José dos Campos School of Dentistry, São Paulo State University (UNESP), São José dos Campos 12245-000, SP, Brazil; sergio.lopes@unesp.br; 6 Postgraduate Program in Dentistry, Cruzeiro do Sul University (UNICSUL), São Paulo 08060-070, SP, Brazil; alfcosta@gmail.com; 7 Department of Dentomaxillofacial Radiology, Faculty of Dentistry, Ankara University, Ankara 06560, Türkiye

**Keywords:** airway remodeling, fluid dynamics, maxillary expansion, maxillary sinus, upper airway

## Abstract

**Background/Objectives:** The effect of rapid maxillary expansion (RME) on the nasal and pharyngeal airways in children remains uncertain. This retrospective study utilized computational fluid dynamics (CFD) to assess the changes in ventilation parameters caused by RME in children. **Methods:** Pre- and post-RME cone beam computed tomography (CBCT) images of 20 patients (4 males, mean age 13 ± 2 years) treated with RME for maxillary transverse insufficiency were evaluated. The RME treatment was conducted using two distinct techniques: tooth-borne and tooth-bone-borne. CFD simulations were used to investigate the airflow conditions (pressure and velocity) in the whole upper airway, nasal airway, and maxillary sinus. Morphological alterations and variations in ventilation parameters before and after RME treatment were statistically compared. The extent of changes in the morphological and ventilatory characteristics of the upper airway, depending on the type of RME, was assessed. Additionally, changes in the ventilation conditions of the upper airway, nasal airway, and maxillary sinus after RME treatment were statistically analyzed. Statistical analyses using IBM SPSS v22 (New York, USA) software included paired *t*-tests, Mann–Whitney U tests, Wilcoxon matched-pairs signed-rank tests, intraclass correlation coefficients, and coefficients of variation (*p* < 0.05). **Results:** The CFD study revealed a notable reduction in both air flow velocity and pressure after the RME treatment (*p* < 0.05). A statistically significant increase was seen in the parameters used to assess the morphological changes following RME treatment, including nasal width, anterior and posterior nasal cross-sectional area, intermaxillary and intermandibulary molar width, and oropharyngeal airway width (*p* < 0.05). Regarding the change in airway ventilation, there was no statistically significant difference between tooth-borne and tooth-bone-borne RME treatments (*p* > 0.05). **Conclusions:** RME not only treats orthodontic issues in childhood but also increases airflow, which enhances ventilation. CDF is an effective method for the detection of ventilation improvement.

## 1. Introduction

One of the most prevalent developmental issues in the maxillofacial region is maxillary transverse insufficiency [1]. Rapid maxillary expansion (RME) is a favored treatment for juvenile patients aimed at augmenting the transversal development of the maxilla [1,2]. Studies have demonstrated that enlarging the transverse dimension of the maxilla results in a larger nasal anatomy [3,4]. This dimensional increase in nasal anatomy has been reported in the literature to have positive effects on nasal airflow resistance, nasal breathing, recurrent ear or adenoid infections, obstructive sleep apnea (OSA), and voice quality [5,6,7]. Centirelli et al. [6], in their study providing an otolaryngological evaluation of RME treatment, demonstrated that RME is not only an orthodontic treatment method but also a therapy that can enhance nasal function, reduce the need for ventilation tube placement, mitigate adeno-tonsillar hypertrophy, and thereby positively impact conductive hearing loss and obstructive sleep apnea.

There are various types of RME devices, each designed differently [8]. In rhinometric comparisons between tooth-borne RME and tooth-bone-borne hybrid RME, the hybrid RME treatment demonstrated higher nasal airflow values and a greater reduction in nasal resistance compared to the tooth-borne RME treatment [9]. Krüsi et al. [10], in their systematic review comparing the effects of bone-borne and tooth-bone-borne hybrid RME treatments with tooth-borne RME, stated that although studies in the literature suggest that RME treatment can improve parameters such as nasal cavity width, nasal airflow, and upper airway volume and reduce nasal airway resistance, the effects of these changes on breathing and quality of life remain uncertain. They emphasized the need for further research in this area [10].

Guijarro-Martínez and Swennen [11] defined the upper airway (UA) as the region from the anterior nostrils to the 4th cervical vertebra in the sagittal plane. They further categorized it into four distinct sections as follows: the nasal cavity, nasopharynx, oropharynx, and hypopharynx [11]. The UA is a complex anatomical region consisting of both soft and hard structures. The morphological properties of the airway have a direct impact on the characteristics of airflow during respiratory activity [12].

Computational fluid dynamics (CFDs) is a discipline within fluid mechanics that uses numerical methods and algorithms to analyze and resolve fluid materials’ flow issues [13]. This technique simulates the interaction between liquids and gases and the surfaces they come into contact with. It computes flow parameters, including flow pressure and velocity. CFD analysis produces airflow that is not influenced by the airway’s shape and simulates the ventilation conditions of the UA in a computerized setting, mimicking the actual breathing process [14]. Suga et al. [15] demonstrated that the use of CFD analysis to measure airflow velocity may successfully identify areas of ventilation disorder in the airway of individuals with sleep apnea. Iwasaki et al. [2] assessed the nasal airway (NA) ventilation of patients undergoing RME treatment using CFD and concluded that this approach was superior to other traditional methods in evaluating respiratory conditions of the NA.

This study aimed to assess the morphological and functional alterations of the airway following RME treatment and determine the extent of changes in UA ventilation through the use of CFD analysis.

## 2. Materials and Methods

The retrospective study underwent assessment and approval by the Ethics Committee of Health Sciences (No. 22.09.2021-14) of Ankara Yildirim Beyazit University, Ankara, Turkiye. It was carried out in compliance with the principles outlined in the Declaration of Helsinki. Due to the study’s retrospective character, the requirement for obtaining informed consent was waived.

The previous investigation on nasal airway velocity (V_NA_) following RME treatment reported a standard deviation (σ) ranging from 11.3 to 21.6 [16]. Consequently, for the present study, a value of 16.45 was used. In addition, it was assumed that the Z value and the effect size were 1.96 and 8, respectively, for the 0.05 type I error rate. According to this sample size equation, the minimum required sample size was determined to be 16.34 (≅16).

This study utilized pre- and post-treatment cone beam computed tomography (CBCT) images of 20 patients who were admitted to the Department of Orthodontics at Eskisehir Osmangazi University between October 2017 and January 2021. These patients were diagnosed with transverse maxillary insufficiency and underwent RME treatment. The sample consisted of 4 male and 16 female participants, with an average age of (13 ± 2) years.

The inclusion criteria were individuals without any craniofacial or developmental abnormalities, no history of prior orthodontic treatment, and patients who had undergone orthodontic treatment resulting in roughly 5 mm of maxillary expansion as determined by measuring the distance between the mesiopalatinal tubercles of the maxillary first molars in diagnostic models. The study group consisted of CBCT images of 20 patients who underwent RME treatment with Hyrax (tooth-borne) (*n* = 8) and Hybrid-Hyrax (tooth and bone-borne) (*n* = 12) expanders.

The initial Cone Beam Computed Tomography (CBCT) images taken prior to treatment (T0) and the CBCT images obtained after the removal of the expander (T1), along with traditional orthodontic diagnostic records, were utilized from the patients. The following procedure was implemented during the treatment of the patients: Both groups had bands placed on their maxillary permanent molars and premolars. The Hybrid-Hyrax group was fitted with bands only on the first maxillary permanent molars, as well as 2 LOMAS mini-implants (7 × 2 mm) (Mondeal, Mühlheim, Germany) bilateral to the mid-palatal suture, positioned at the level of the third palatal rugae. The appliances in the Hyrax and Hybrid-Hyrax groups were assembled by soldering a standard activation screw (Hyrax, Dentaurum, Ispringen, Germany). Cementation was performed using glass ionomer cement (Unitek™ Multi-Cure Glass Ionomer Orthodontic Band Cement, 3M Oral Care, Maplewood, MN, USA). The activation protocol consisted of two turns every day (0.5 mm). Weekly follow-up sessions were scheduled until the posterior crossbite was overcorrected, which means that the palatal cusps of the maxillary first molars were in contact with the buccal cusps of the mandibular first molars. Retainers were left in place as retention devices without additional orthodontic treatment for 6 months.

The images used in this study were obtained on a Planmeca Promax Mid (Helsinki, Finland) using the standard imaging procedure (84 kVp, 6 mA, 8.5 s, Fov 20 × 17 cm). The patients were placed in a position with the Frankfort horizontal plane parallel to the floor, teeth in centric occlusion, and the tongue at rest, with no swallowing or soft tissue movement. Images were then taken in standardized settings. The images were stored in the digital imaging and communications in medicine (DICOM) format. The CBCT images included in the research group were chosen from images with a craniocervical inclination of 95–105°, possessing high diagnostic quality, and providing enough visualization of the patient’s anatomical features in detail [17].

Cephalometric angle values, reference planes, and anatomical landmarks were derived from cephalometric images produced from CBCT images [18]. The parameters and abbreviations used to evaluate the morphological changes caused by RME treatment are shown in Table 1.

**Figure 1:**
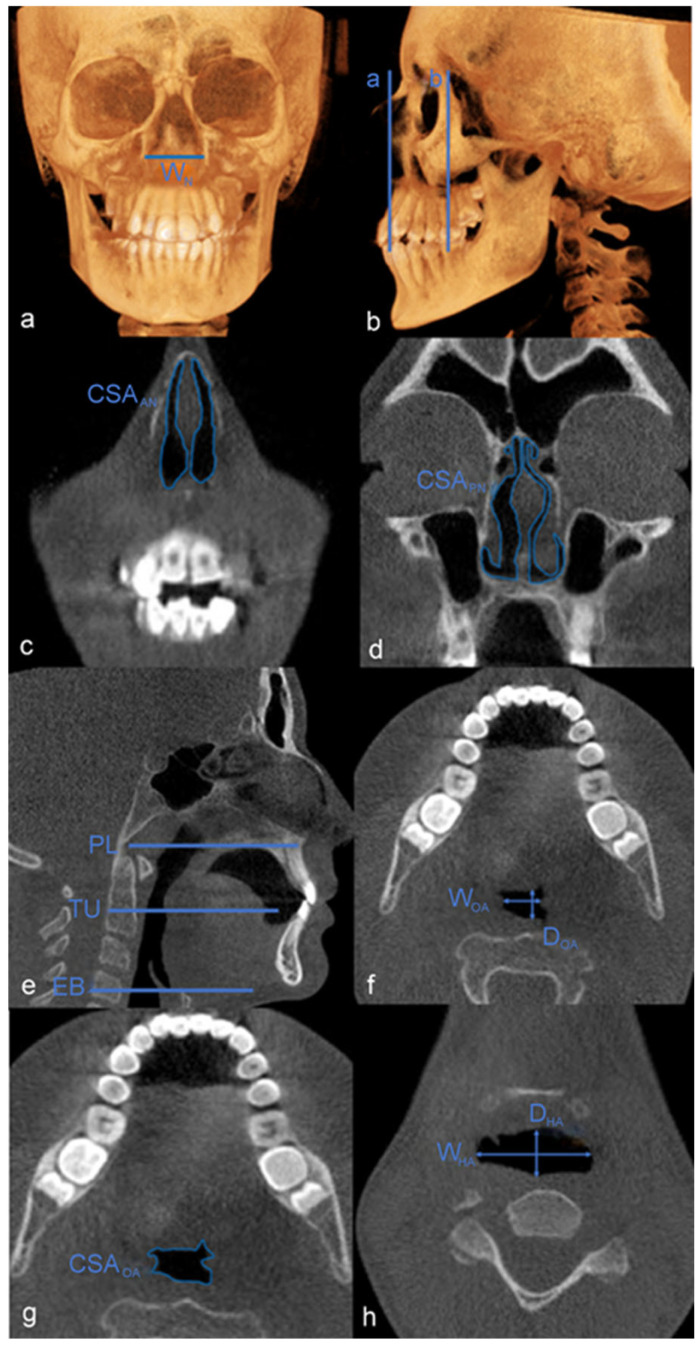
Morphological measurement regions: (**a**) W_N_, (**b**) a. CSA _AN_ measurement level and b. CSA_PN_ measurement level, (**c**) CSA_AN_, (**d**) CSA_PN_, (**e**) Oropharyngeal, hypopharyngeal measurement level (PL: palatal plane, TU: tip of uvula, EB: base of epiglottis); (**f**) W_OA_ and D_OA_, (**g**) CSA_OA_, and (**h**) W_HA_ and D_HA._.

These measurements were obtained from CBCT images and 3D reconstruction images before and after treatment [2,14,18] (Table 1 and Figure 1). Each measurement was taken at two time points as follows: prior to treatment (T0) and after the completion of active expansion (T1). Since the sample size was small in groups, airway comparison according to malocclusion was not performed.

Approximately 250 axial and 160 sagittal sections from each patient’s CBCT data were analyzed to obtain anatomically accurate 3D models of the UA. The 3D models were generated using threshold segmentation using the 3D medical image processing software Mimics Innovation Suite 23.0 (Materialize, Leuven, Belgium). Consequently, the threshold value was established within the range of −500 to −1024 Hounsfield units to differentiate between soft tissue and an open airway [15]. A 3D model of the airway, spanning from the frontal sinus to the 3rd cervical vertebra, was generated by identifying and including all the spatial regions inside the airway region. The 3D models were imported into the 3-matic Version 15 (Materialise, Leuven, Belgium) in the stereolithography format. Following the mesh morphing procedure, the 3D models of the UA were modified to be appropriate for a CFD study without any loss of data.

**Figure 2:**
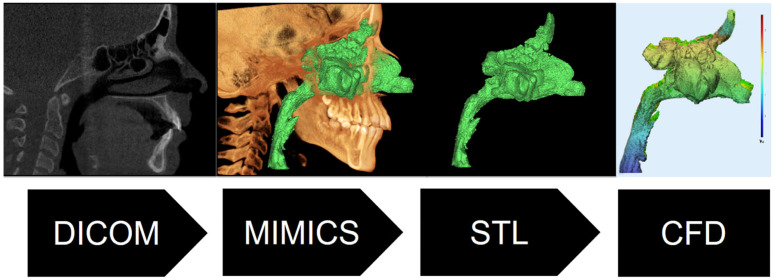
CFD analysis workflow.

The 3D models of the upper airway (UA), nasal airway (NA), and maxillary sinus (MS) were imported into SolidWorks Flow Simulation (Dassault Systèmes, v 2022, Vélizy, Villacoublay, France) for CFD analysis (Figure 2). The meshing process was performed using an adaptive tetrahedral mesh generator, with a global element size of 0.5 mm and local refinement (0.2 mm) applied near the airway walls to resolve boundary layer effects. The final mesh comprised approximately 3–5 million elements per model, ensuring a y+ value of <5 for accurate turbulence modeling. The y+ value is a dimensionless parameter used in CFD to assess the resolution of the near-wall mesh in turbulence modeling.

The airflow was simulated as steady-state, incompressible, and turbulent, governed by the Reynolds-averaged Navier–Stokes (RANS) equations. The standard κ−ε turbulence model was selected due to its validated performance in simulating physiologically relevant airway flows [13,19,20]. Turbulence intensity at the nostrils was set to 5%, with a hydraulic diameter of 10 mm based on average patient anatomy [19]. Boundary conditions are included as follows:

- Velocity inlets: Bilateral nostrils with a volumetric flow rate of 200 mL/s, corresponding to resting breathing conditions [18].
- Pressure outlet: Hypopharynx plane set to 0 Pa (atmospheric pressure) [18,19].
- Wall conditions: Rigid, no-slip surfaces with adiabatic properties.

The solver utilized a second-order upwind discretization scheme for momentum and turbulence equations. Simulations were iterated until residuals for continuity, momentum, and turbulence parameters fell below 0.2%, ensuring convergence. The pressure drop and maximum airflow velocity within the UA were measured and evaluated at two distinct time points, denoted as t0 and t1 [19]. Additional methodological details align with prior studies evaluating UA airflow [2,14,19]. 

The adenoid tissues, which might potentially cause obstruction in the UA, were assessed in the midsagittal plane using CBCT images. They were categorized into four categories, following the methodology employed by Major et al. [21] in their comparative research, including nasoendoscopy. The tonsillar tissues were evaluated in the coronal plane of the oropharyngeal airway’s narrowest part and classified into five groups [18] (Table 1).

### 2.1. Statistical Analysis

Descriptive statistics of the study group based on age, gender, and type of RME treatment were performed. A paired *t*-test was utilized to compare the morphological alterations and ventilation parameters before and after RME treatment. The Mann–Whitney U test was employed to assess the amount of alteration in morphological and ventilation characteristics of the UA based on the type of RME. The Spearman correlation coefficients were computed to assess the associations between W_N_, W_U6_, and W_OA_ variables and the conditions of total UA, NA, and maxillary sinus (MS) ventilation. The data collected in this study were analyzed using the IBM SPSS Statistics V 22.0 (New York, USA) package program. The study data were evaluated using descriptive statistical methods, including the mean, standard deviation, and *p*-value. A significance level of 0.05 was employed, and it was indicated that there was a statistically significant difference or correlation at *p* < 0.05.

### 2.2. Examiner Reliability

All morphological measurements were taken twice by the two observers (A.A., K.O.), and the mean values of all measurements were included in the statistical analysis. The observers also performed the measurements twice with an interval of 1 month to detect intra-observer variability. Moreover, before starting the radiographic examination in the study, the examiners were calibrated to recognize as well as to identify the UA anatomy. For such a purpose, a different 5 CBCTs, other than this study, were used. The examiners only examined the CBCTs and were blinded to any other patient data in the radiographic examination procedure. Measurements were repeated to evaluate the CSA_AN_, CSA_PN_, W_OA_, D_OA_, CSA_OA_, W_HA_, D_HA_, and CSA_HA_ for both inter- and intra-observer reliability.

To assess intra-observer reliability, the Wilcoxon matched-pairs signed rank test was used for repeat measurements. The inter-observer reliability was determined by the intraclass correlation coefficient (ICC) and the coefficient of variation (CV) [CV = (standard deviation/mean) × 100%]. Values for the ICC range from 0 to 1. ICC values greater than 0.75 show good reliability, and the low CV demonstrates the precision error as an indicator for reproducibility [22].

## 3. Results

### 3.1. Intra-Observer Consistency

Repeated CBCT evaluation and measurements indicated no significant intra-observer difference for both observers (*p* > 0.05). Overall intra-observer consistency for observer 1 was rated at 88% and 92%, while the consistency for observer 2 was found at 89% and 91% between the two evaluations and measurements, respectively. All measurements were found to be highly reproducible for both observers, and no significant difference was obtained between the two measurements of the observers (*p* > 0.05).

### 3.2. Inter-Observer Consistency

The ICCs between Observer 1 and Observer 2 ranged from 0.881 to 0.962. There was a high inter-observer agreement, while a high ICC and low CV demonstrated that the procedure was standardized between the evaluations and measurements of the observers. No statistical differences were found among observers evaluations and measurements (*p* < 0.05). Observer 1 had the highest intra-observer consistency; thus, the mean of this observer’s evaluations and measurements was chosen for further analysis.

The present study assessed UA ventilation by CFD analysis using CBCT images acquired before and after treatment in a cohort of 20 patients, including 4 males, who had RME treatment. The average age of this sample was 13 (±2) years. The patients received RME treatment using two distinct techniques as follows: tooth-borne (*n* = 8) and tooth-bone-borne (*n* = 12). This study investigated alterations in the structure and airflow dynamics of the UA.

Following RME treatment, there were notable and statistically significant enhancements in various parameters. These included an increase in W_N_, W_U6_, W_L6_, and W_OA_; an expansion of CSA _AN_ and CSA_PN_; and an improvement in the SNA°, the ANB°, and the FMA° (Table 2). These modifications led to the conclusion that there was an increase in size in the nasal and oropharyngeal areas following RME treatment.

**Figure 3:**
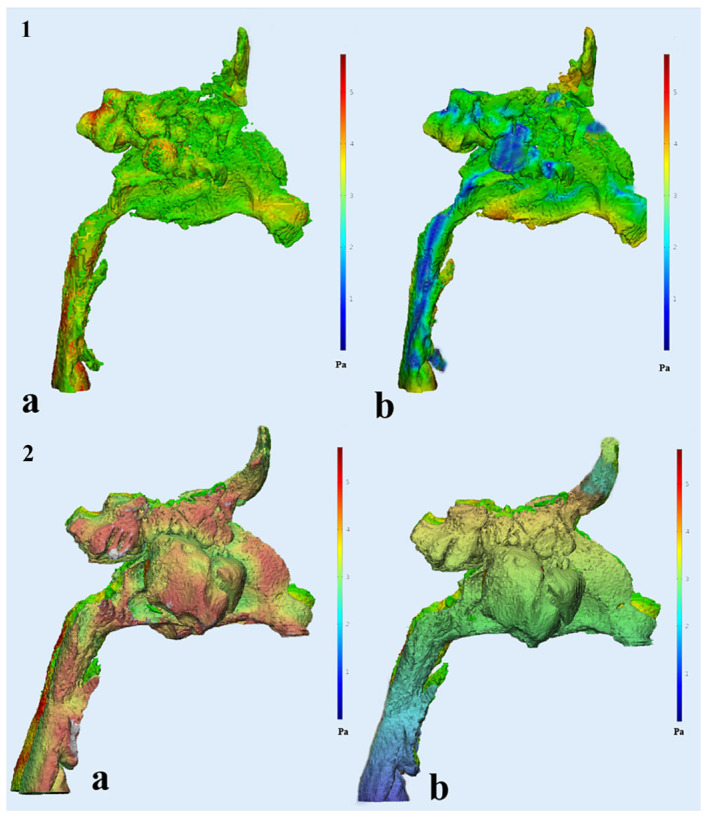
Demonstration of ventilation changes following RME treatment with CFD: (**1**) tooth-borne RME treatment—(**a**) pre-treatment P_UA_ distribution and (**b**) post-treatment P_UA_ distribution; (**2**) tooth-bone-borne RME treatment—(**a**) pre-treatment P_UA_ distribution and (**b**) post-treatment P_UA_ distribution; The red color code represents the increasing value, while the blue color code represents the decreasing value.

The airway pressure (P_A_) and airway velocity (V_A_) characteristics following RME treatment were examined using CFD analysis. After the treatment, there were significant reductions in airway parameters. These included P_UA_ (−99.6 Pa), V_UA_ (−3.06 m/s), P_NA_ (−18.59 Pa), V_NA_ (−4.53 m/s), P_MS_ (−31.64 Pa), and V_MS_ (−3.4 m/s) (Table 2). While there was no notable enlargement in the size of the UA in terms of volume and surface area, there was a remarkable enhancement in the efficiency of airway ventilation (Figure 3).

Comparing the post-treatment morphological and ventilation changes in the UA resulting from two different methods of RME, it was found that patients who underwent tooth-borne RME treatment had significantly less nasal expansion (*p* < 0.05) compared to those who underwent tooth and bone-borne RME treatment. The variation in treatment approach did not result in a statistically significant disparity in the other assessed parameters (Table 3).

Following RME treatment, there was a considerable increase in W_N_ (mm), W_U6_ (mm), W_L6_ (mm), CSA_AN_ (mm^2^), CSA_PN_ (mm^2^), and W_OA_ (mm). However, no correlation was found between these measurements and the ventilation parameters that were examined.

The Wilcoxon rank test evaluated alterations in the dimensions of adenoid and tonsillar tissue pre- and post-RME treatment. The post-treatment alteration in the size of adenoid and tonsillar tissue did not demonstrate any statistically significant difference.

Regardless of the physical size of the adenoid tissue and tonsillar hyperplasia that cause airway obstruction in patients, an enhancement in UA ventilation was seen using CFD analysis conducted following RME treatment.

## 4. Discussion

Multiple studies in the literature demonstrate that RME treatment effectively enhances airway ventilation and can be considered a viable therapeutic option for situations of increased airway resistance, particularly when there is an obstruction originating from the nasal region [2,23]. Nevertheless, Haight and Cole [24] showed in their research that in two-thirds of patients experiencing complete nasal resistance, the obstruction was caused by an enlargement in the nasal valve area and conchae. Sulsenti and Palma [25] have reported that the nasal valve region shows the greatest nasal resistance, and even minor abnormalities in this area significantly impact the flow dynamics of the NA. Enoki et al. [26] reported that RME treatment improved nasal respiration but was not a treatment option for the treatment of airway obstruction as mucosal changes were much smaller than bony changes. Zawiślak et al. [27] utilized the widths of the second maxillary molar, the base of the maxilla, and the pyriform aperture at the base of the inferior concha as criteria for evaluating skeletal changes in their study, which examined dental and skeletal alterations in patients with transverse maxillary deficiency treated with transpalatal distraction. They reported that the increase in the width of the pyriform aperture at the base of the inferior concha following treatment was perceived by patients as an improvement in nasal patency [27]. Zyła et al. [28] reported that, in patients with transverse maxillary insufficiency, when the SARME procedure was performed without including pterygoid disjunction, a V-shaped opening was observed in the maxilla, similar to the effect of RME applied with the Hyrax expander. This resulted in an increase in the skeletal structure of the anterior maxilla in the transverse direction [28]. Although numerous studies in the literature demonstrate the effects of RME on nasal and maxillary expansion, the impact of RME on reducing nasal resistance remains a subject of debate [6]. Our study did not find any statistically significant changes in most of our morphological measures after undergoing RME treatment, except for W_N_ (mm), W_U6_ (mm), W_L6_ (mm), CSA_AN_ (mm^2^), CSA_PN_ (mm^2^), and W_OA_ (mm) values. However, statistically significant changes in pressure and velocity variables obtained as a result of ventilation simulation with CFD and improvements in ventilation conditions were observed. It is anticipated that there will be an increase in airway ventilation due to morphological alterations. Nevertheless, the assessment of ventilation using CFD has revealed that even minor morphological alterations in the airway, which may not be statistically significant, might enhance ventilation. Our study yielded results that align with the existing literature about the enhancement of ventilation due to RME treatment and its detection by CFD simulation [2,26].

The impact of airway dimension enlargement on alterations in ventilation conditions is a topic of ongoing controversy and study [11]. Upon reviewing the literature and assessing the findings of our study, we concluded that employing a functional method to evaluate the results of RME treatment would yield more precise outcomes. Our findings revealed a statistically significant dimensional augmentation in bone structure subsequent to RME treatment (Table 2). For the functional evaluations, we utilized CFD simulation to assess the UA and NA. The results showed a substantial reduction in both the airflow pressure and velocity in the UA as well as in the NA after treatment (Table 2). Nevertheless, there was no statistically significant correlation between the increase in the size of the bone structure in the airway and the improvement in ventilation conditions. A significant statistical difference was seen in the outcomes of the airway ventilation functional assessment before and after treatment. These findings demonstrate that utilizing the CFD analysis approach yields more precise data on the impact of RME treatment on ventilation conditions. Iwasaki et al. [2] obtained similar results to our study in their study in which they evaluated the NA ventilation of patients undergoing RME treatment using CFD. The researchers asserted that relying solely on morphological data for evaluating the NA is inadequate due to its intricate composition of soft tissue and bone structures, which are influenced by atmospheric conditions. They stated that conducting CFD simulations provides more precise results for the functional assessment of the NA [2].

In their study, Iwasaki et al. [7] evaluated changes in nasal ventilation parameters following RME treatment using conventional tooth-borne Hyrax, tooth-bone-borne Hybrid-Hyrax, and Keles keyless expanders, utilizing CBCT data and CFD analysis. They found that Hybrid-Hyrax was more effective than both Hyrax and Keles in increasing nasal width and CSA and in reducing airway pressure and velocity; however, these differences did not reach statistical significance [7]. In our study, Hyrax and Hybrid-Hyrax expanders were compared. It can be concluded that Hybrid-Hyrax expanders provide greater benefits, as they demonstrated statistical significance in increasing the width of the nasal cavity. However, for the other parameters evaluated, no significant differences were observed regarding the effect of the expanders on treatment, which aligns with the findings of Iwasaki et al. [7] (see Table 3).

Sakoda-Iwata et al. [16] assessed the ventilation conditions of children with nasal mucosa hypertrophy and adenoid obstructions following RME treatment using CFD. The study found that NA obstruction significantly improved following RME treatment in children who did not have nasal mucosa hypertrophy or obstructive adenoids. However, the amount of improvement in ventilation was reduced in individuals with obstructive adenoids and nasal mucosa hypertrophy. Furthermore, NA obstruction associated with obstructive adenoids did not show any improvement [16]. When assessing the ventilation conditions of the UA, it is important to consider the presence of adenoid tissues, tonsils, and any alterations in the mucosa. Our investigation found that the use of RME treatment did not result in a statistically significant alteration in the size of adenoid tissues and tonsils. Nevertheless, the CFD study demonstrated a statistically significant augmentation in airway ventilation. This finding corroborates the view that even the bone structure and mucosal changes that occur with RME treatment cause improvements in airway ventilation.

The whole of the UA, spanning from the nostrils to the base of the epiglottis, was assessed in our study. Additionally, we examined specific regions, such as the NA and MS. Several studies in the literature have assessed the ventilation conditions in the paranasal sinuses using CFD after surgical interventions in the paranasal or nasal area [29,30]. In this study, we observed that the millimetric opening of the palatal suture with RME treatment significantly improved the airflow pressure (*p* < 0.001) and velocity (*p* < 0.001) in the MS, as determined by CFD analysis.

In CBCT research conducted by Zhao et al. [31], the oropharyngeal airway volume was evaluated in 12-year-old children who had RME treatment. They did not detect an increase in oropharyngeal airway volume following the treatment. Iwasaki et al. [32] assessed the alteration in tongue posture and pharyngeal airway following RME treatment and concluded that the treatment had no effect on the volume of the airway. In our study, we observed a noteworthy augmentation in the W_OA_ following RME treatment. However, we did not find any statistically significant increase in the cross-sectional area, volume, or surface area of the UA.

One of the limitations of our study is the small sample size. Due to the retrospective design of our investigation, it was performed with a limited number of patients with CBCT data suitable for this study. In addition, the data obtained by CFD simulation could not be confirmed by polysomnography or rhinomanometry due to the lack of a clinical study. Prospective studies with a greater number of participants and clinically verifiable methods will provide a clearer indication of the clinical effectiveness of CFD simulation in evaluating airway ventilation.

## 5. Conclusions

In conclusion, when it comes to alterations in airway airflow, tooth-borne and tooth-bone-borne RME therapies have comparable outcomes. Clinical assessment and traditional procedures can be used to evaluate the airway comprehensively. CFD simulation gives the option to evaluate the airway as a whole or in portions, giving a more accurate and focused assessment of the effect of the treatment.

## Figures and Tables

**Table 1:** Parameters used in this study.

Abbreviations	Definitions	Descriptions	Images Utilized
S	Sella turcica	Midpoint of the sella turcica	Cephalometric images generated from CBCT
N	Nasion	Intersection between the internal and nasofrontal sutures in the midsagittal plane	Cephalometric images generated from CBCT
A	A point	Deepest midline point of the premaxilla between the anterior nasal spine and prosthion	Cephalometric images generated from CBCT
B	B point	Posterior point of the concavity between infradentale and pogonion	Cephalometric images generated from CBCT
PL	Palatal Plane	A plane parallel to the hard palate, passing through the posterior nasal spine	CBCT multiplanar reformation (MPR)
Vol_UA_ (cm^3^)	Upper airway volume	From the anterior nostrils to the 3rd cervical vertebra	3D models segmented from CBCT
SA_UA_ (cm^2^)	Upper airway surface area	From the anterior nostrils to the 3rd cervical vertebra	3D models segmented from CBCT
P_UA_ (Pa)	Upper airway pressure	From the anterior nostrils to the 3rd cervical vertebra	3D models segmented from CBCT
V_UA_ (m/s)	Upper airway velocity	From the anterior nostrils to the 3rd cervical vertebra	3D models segmented from CBCT
P_MS_ (Pa)	Maxillary sinus pressure	Maxillary Sinus Region	3D models segmented from CBCT
V_MS_ (m/s)	Maxillary sinus velocity	Maxillary Sinus Region	3D models segmented from CBCT
P_NA_ (Pa)	Nasal airway pressure	From the anterior nostrils to the line extending from Sella to the PNS	3D models segmented from CBCT
V_NA_ (m/s)	Nasal airway velocity	From the anterior nostrils to the line extending from Sella to the PNS	3D models segmented from CBCT
W_N_ (mm)	Nasal width	The widest portion of the nasal aperture	CBCT 3D reconstruction
W_U6_ (mm)	Intermaxillary molar width	The intermaxillary molar width at the narrowest portion	CBCT 3D reconstruction
W_L6_ (mm)	Intermandibulary molar width	The intermandibulary molar width at the narrowest portion	CBCT 3D reconstruction
CSA_AN_ (mm^2^)	Anterior nasal cross-sectional area	The level of the anterior nasal spine	CBCT MPR
CSA_PN_ (mm^2^)	Posterior nasal cross-sectional area	15 mm posterior to the anterior nasal spine	CBCT MPR
W_OA_ (mm)	Oropharyngeal airway width	The narrowest portion of the oropharyngeal airway cross-section	CBCT MPR
D_OA_ (mm)	Oropharyngeal airway depth	The widest portion of the oropharyngeal airway cross-section	CBCT MPR
CSA_OA_ (mm^2^)	Oropharyngeal airway cross-section area	Measured along the PL plane passing through the tip of the uvula	CBCT MPR
W_HA_ (mm)	Hypopharyngeal airway width	The narrowest portion of the hypopharyngeal airway cross-section	CBCT MPR
D_HA_ (mm)	Hypopharyngeal airway depth	The widest portion of the hypopharyngeal airway cross-section	CBCT MPR
CSA_HA_ (mm^2^)	Hypopharyngeal airway cross-section area	Measured along the PL plane passing through the base of the epiglottis	CBCT MPR
L_H_ (mm)	Hyoid length	PL to the most superoanterior point of the hyoid	CBCT MPR

**Table 2:** The ventilation and morphological changes seen with RME treatment, mean (SD).

	Scanning Times		Paired *t*-Test
	T0 (*n* = 20)	T1 (*n* = 20)	*p*
P_UA_ (Pa)	383.90 (46.91)	284.29 (43.63)	<0.001
V_UA_ (m/s)	19.25 (3.16)	16.19 (3.48)	<0.001
P_MS_ (Pa)	85.78 (23.44)	54.14 (10.76)	<0.001
V_MS_ (m/s)	13.26 (2.93)	9.90 (3.03)	<0.001
P_NA_ (Pa)	81.49 (7.94)	62.90 (8 73)	<0.001
V_NA_ (m/s)	12.09 (1.25)	7.56 (1.25)	<0.001
SNA (°)	80.58 (3.96)	81.52 (4.67)	0.026
ANB (°)	2.86 (2.63)	4.27 (2.58)	<0.001
FMA (°)	25.23 (4.29)	27.04 (4,73)	0.002
W_N_ (mm)	22.79 (1.74)	25.24 (1.80)	<0.001
W_U6_ (mm)	32.36 (3.13)	36.45 (3.48)	<0.001
W_L6_ (mm)	32.12 (3.08)	32.91 (2.57)	0.032
CSA _AN_ (mm^2^)	203.69 (38.38)	233.31 (48.42)	0.045
CSA_PN_ (mm^2^)	304.40 (58.40)	327.17 (80.97)	0.002
W_OA_ (mm^2^)	16.24 (4.95)	18.81 (6.34)	0.012

*n*: number of participants; SD: standard deviation.

**Table 3:** Ventilation and morphological changes due to Hyrax and Hybrid-Hyrax RME treatments, mean (SD).

	Difference Between T1–T0		Mann–Whitney U Test
	Hyrax (*n* = 8)	Hybrid-Hyrax (*n* = 12)	*p*
P_UA_ (Pa)	−99.73 (11.07)	−99.65 (23.48)	0.64
V_UA_ (m/s)	−2.70 (2.3)	−3.30 (3.27)	0.76
P_MS_ (Pa)	−25.46 (11.61)	−35.76 (14.26)	0.11
V_MS_ (m/s)	−3.18 (3.15)	−3.48 (2.09)	0.88
P_NA_ (Pa)	−19.67 (7.94)	−17.88 (7.48)	0.59
V_NA_ (m/s)	−4. 56 (0.90)	−4.17 (0.72)	0.192
SNA (°)	0.76 (0.96)	1.05 (2.13)	0.616
SNB (°)	−1.30 (1.87)	0.12 (1.16)	0.07
ANB (°)	2.06 (1.49)	0.98 (1.32)	0.064
FMA (°)	2.50 (2.60)	1.35 (2.01)	0.70
W_N_ (mm)	1.40 (1.07)	3.15 (1.89)	0.037 *
W_U6_ (mm)	5.33 (2.45)	3.26 (2.76)	0.105
W_L6_ (mm)	0.18 (1.39)	1.21 (1.55)	0.097
CSA _AN_ (mm^2^)	9.73 (36.17)	42.89 (48.72)	0.064
CSA_PN_ (mm^2^)	1.98 (78.10)	36.62 (48.76)	0.396
W_OA_ (mm)	4.23 (4.74)	1.47 (3.48)	0.28
D_OA_ (mm)	21.73 (2.77)	22.60 (5.31)	0.537
CSA_OA_ (mm^2^)	50.65 (33.73)	4.13 (86.53)	0.165
W_HA_ (mm)	0.12 (1.72)	0.58 (1.81)	0.616
D_HA_ (mm)	− 0.01 (1.18)	− 0.2 (2.54)	0.70
CSA_HA_ (mm^2^)	−3.56 (35.63)	10.20 (61.93)	0.44
L_H_ (mm)	2 (5.45)	1.91 (4.66)	0.758
Vol_UA_ (cm^3^)	9500.75 (14,537.11)	226.25 (7137.88)	0.105
SA_UA_ (cm^2^)	2949.01 (4727.14)	454 (3226.80)	0.316

*n*: number of participants; SD: standard deviation; * *p* < 0.05.

## Data Availability

The raw data supporting the conclusions of this article are available from the corresponding author on reasonable request.

## Abbreviations

The following abbreviations are used in this manuscript:

**Abbreviations**	**Definitions**
S	sella turcica
N	nasion
A	A point
B	B point
PL	palatal plane
Vol_UA_ (cm^3^)	upper airway volume
SA_UA_ (cm^2^)	upper airway surface area
P_UA_ (Pa)	upper airway pressure
V_UA_ (m/s)	upper airway velocity
P_MS_ (Pa)	maxillary sinus pressure
V_MS_ (m/s)	maxillary sinus velocity
P_NA_ (Pa)	nasal airway pressure
V_NA_ (m/s)	nasal airway velocity
W_N_ (mm)	nasal width
W_U6_ (mm)	intermaxillary molar width
W_L6_ (mm)	intermandibulary molar width
CSA _AN_ (mm^2^)	anterior nasal cross-sectional area
CSA_PN_ (mm^2^)	posterior nasal cross-sectional area
W_OA_ (mm)	oropharyngeal airway width
D_OA_ (mm)	oropharyngeal airway depth
CSA_OA_ (mm^2^)	oropharyngeal airway cross-section area
W_HA_ (mm)	hypopharyngeal airway width
D_HA_ (mm)	hypopharyngeal airway depth
CSA_HA_ (mm^2^)	hypopharyngeal airway cross-section area
L_H_ (mm)	hyoid length
